# Do It Once: Concatenating the Image Pair for a Single Pass Feature Extraction in Stereo Depth Sensing

**DOI:** 10.3390/s26123919

**Published:** 2026-06-20

**Authors:** Žan Regoršek, Andrej Žemva

**Affiliations:** Faculty of Electrical Engineering, University of Ljubljana, 1000 Ljubljana, Slovenia; zr1677@student.uni-lj.si

**Keywords:** stereo vision, depth estimation, real-time inference, feature extraction, edge computing

## Abstract

**Highlights:**

**What are the main findings?**

- A single-pass feature extraction approach, based on concatenating stereo image pairs, improves inference speed by 10–39% across multiple state-of-the-art models.
- The proposed method preserves accuracy while introducing only a moderate increase in runtime memory usage, with no changes to the underlying network architecture.

**What are the implications of the main findings?**

- Stereo depth models can be significantly accelerated through simple plug-and-play modifications to the feature extraction module on existing or future deep-learning-based stereo depth models, with demonstrated benefits on both high-performance GPU and edge devices.

**Abstract:**

In the field of stereo depth sensing, modern research predominantly prioritizes accuracy, yet inference speed remains a critical bottleneck for practical, real-time applications on resource-constrained platforms. Existing acceleration approaches often rely on lighter network architectures or runtime-specific optimizations, which may require architectural redesign, platform-specific tuning, or accuracy trade-offs. However, a common inefficiency remains in many stereo pipelines: feature extraction is typically performed using two separate forward passes, one for the left image and one for the right, even though both passes use the same network weights. We address this redundancy by concatenating the left and right images into a single combined tensor, enabling feature extraction in one batched pass while preserving the original network architecture. By reducing feature extraction time by up to 48.4%, our results demonstrate that this method accelerates the overall inference rate by 10% to 39% on average on Nvidia V100 and up to 28.4% on edge device, depending on the model architecture. This speedup is achieved at the expense of only a moderate increase in runtime memory consumption, while retaining the original accuracy. Because the method does not alter the core stereo network, it can be applied as a plug-and-play enhancement to both existing and newly developed stereo matching models.

## 1. Introduction

Autonomous vehicles, humanoid robots, and search-and-rescue rovers require robust perception of their surroundings. Stereo camera systems are widely adopted for depth sensing due to their cost-effectiveness, passive structure, and high accuracy [1,2,3]. While deep-learning-based algorithms achieve remarkable accuracy in depth inference, they are computationally intensive. This presents a major challenge for autonomous devices with limited battery capacity and processing power, creating a critical need for algorithms that are not only accurate but also efficient.

**Figure 1:**
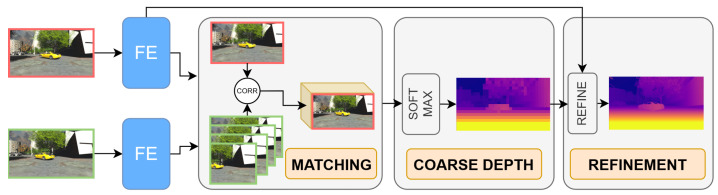
A standard deep learning model for stereo depth inference consisting of a feature extraction network, matching stage, coarse depth estimation stage and refinement stage.

Conceptually, a typical deep-learning-based stereo depth estimation model consists of four stages: feature extraction (FE), feature matching, coarse depth estimation, and depth refinement as illustrated in Figure 1. In the first stage, the three-channel red-green-blue left and right images are encoded into multi-channel feature maps. Subsequently, the right feature map is shifted pixel-by-pixel relative to the left. For every spatial location (pixel) in the left image, a correlation metric is computed against every corresponding pixel offset in the right image. This pixel shift, known as disparity, can be directly translated to depth as defined in Equation (1), where *B* is the baseline, *f* is the focal length, and *d* is the disparity. The correlation metric (often termed cost) at each disparity level can be represented as a scalar or a vector, resulting in a 3D or 4D cost volume (dimensions: width × height × disparity × correlation (scalar or vector)). A Softmax operation is typically applied across the disparity dimension to produce a coarse depth estimate. Since this estimate may be discontinuous and agnostic to context, a final refinement step utilizes features from the left image to enhance the depth map.

(1) $$D=\frac{B\cdot f}{d},$$

One major inefficiency in current stereo pipelines is that FE is typically performed independently for the left and right images. This results in two sequential forward passes through the same network, requiring the network weights to be loaded from memory twice per stereo pair. This redundancy motivates a data-flow transformation in which the two FE passes are replaced by a single combined pass, which reduces total memory traffic and, in turn, computation time.

Deep learning fundamentally transformed stereo depth perception, evolving from early hybrid approaches that applied neural networks solely for feature matching [4], to the first end-to-end trainable models [5], and subsequently to more sophisticated pyramid encoder-decoder architectures [6]. However, maximizing accuracy in these pipelines often relies on 4D cost volumes processed by computationally demanding 3D convolutions, creating a significant performance bottleneck.

To address this, [7] limits the use of 3D convolutions by employing a lightweight 3D aggregation module enhanced with attention, and constructs the cost volume using a combination of group-wise L1 distance and group-wise correlation. Building on a similar idea, [8] reduces reliance on computationally expensive 3D convolutions while maintaining high accuracy through a more expressive cost volume and multi-scale refinement, both enhanced by attention mechanisms that focus on the most relevant information.

Ref. [9] replaces 3D convolutions with a recurrent architecture where the balance between efficiency and accuracy can be adjusted by altering the number of iterative refinement steps. Ref. [10] introduced a geometry encoding volume to accelerate convergence, effectively reducing the necessary iterations. Alternatively, the authors of [11] proposed a combined within-view and cross-view image fusion strategy with confidence-based filtering to improve accuracy, while substituting expensive 3D convolutions with 2D variants for efficiency. Similarly, ref. [12] presented a lightweight architecture by eliminating 3D convolutions entirely, compensating with increased channel width in 2D layers. Feature extraction can account for nearly half the inference time, leading [13] to propose a specialized backbone as an alternative to standard U-Net structures based on MobileNetV2 [14] or EfficientNet [15]. Researchers presented novel backbones such as [16] built specifically for mobile devices, though it is inferior to the above-mentioned backbones as reported in [17] if used in its default state. However, specialized backbones can sometimes be computationally more demanding than networks composed purely of standard MobileNet or EfficientNet blocks, which benefit from extensive optimization in modern inference stacks. Other fusion-based approaches [17,18] compensate for the accuracy loss of lighter model architectures by introducing an additional depth source, such as a Time-of-Flight sensor. However, this strategy requires extra hardware components that are not always available in existing stereo camera setups and increase the cost of new systems.

While existing research primarily focuses on architectural modifications to reduce operation counts or utilize standard operations that are better optimized on GPUs, the cost of data movement is often overlooked. In hardware, memory transfers can dominate execution time as weights must first be fetched from non-volatile memory (e.g., Flash, SSD) to VRAM on GPU, and subsequently forwarded to the execution core cache. Layer-wise execution produces large intermediate feature maps that must be stored in limited VRAM or spilled to system RAM. In memory-constrained environments, this excessive data movement becomes a bottleneck, significantly slowing down execution. Consequently, reducing redundant data movement is a promising but underexplored direction for improving inference efficiency.

To mitigate this, ref. [19] proposed a Depth-First approach where, for a patch of pixels, several successive layers are computed and intermediate values are discarded immediately to reduce memory traffic. Ref. [20] applied this to stereo depth estimation, achieving performance gains of 10–20%. While effective on custom hardware like Field Programmable Gate Arrays (FPGAs), these methods are difficult to deploy on low-cost edge systems using consumer-grade AI accelerators, which rely on specific software stacks (e.g., ONNX [21]) and a fixed library of optimized operators.

Runtime-level concurrency has been explored as a way to improve CNN inference throughput. For example, Huang et al. [22] evaluated CUDA-based strategies such as pipelining, batching, CUDA streams, CUDA contexts, and NVIDIA MPS for running multiple CNN inference workloads concurrently. While such approaches can improve utilization, they rely on platform-specific execution mechanisms and require careful tuning of streams, contexts, transfer modes, and runtime scheduling. This limits their portability to non-CUDA accelerators and alternative inference stacks. Furthermore, conventional batched processing treats batched inputs as independent samples and therefore does not exploit the specific redundancy present in stereo depth neural networks, where the same FE stage is applied separately to the left and right images. Although such batching may improve throughput, it requires multiple input sets to be captured and buffered before inference can begin. In addition, the larger batch increases the amount of data processed in a single inference call. Together, these effects can negatively affect latency and generate output data in bursts which is not desirable in real-time systems that expect continuous, low-latency depth information.

In this work, we seek a universal higher-level optimization for better concurrency and resource utilization, making the approach independent of specific GPU vendors, low-level runtime APIs, or deployment frameworks. Therefore, we propose a simple yet effective approach in the model data flow that concatenates the stereo image pair and performs FE in a single forward pass. This theoretically halves number of memory transactions and kernel launches, and effectively accelerates the overall inference rate by increasing throughput through decreased latency which is especially important for real-time edge devices. Accordingly, our main contributions are:

- We identify redundant left/right FE as a source of inefficiency in stereo matching pipelines and introduce a method that removes this redundancy by processing the stereo pair in a single FE pass.
- We define and evaluate several stereo-pair concatenation strategies, including batch, spatial, and multi-cut variants, and analyze how these alternatives affect inference speed, memory usage, and accuracy.
- We validate the approach across three structurally different stereo networks, showing 10–39% average inference speed improvements while maintaining accuracy.
- We provide TensorRT layer-level profiling to explain where the speedup arises, showing that the gains are primarily driven by reduced FE execution time.
- We demonstrate applicability of our work on an edge device with up to 28.4% FPS increase.

In summary, this work addresses stereo inference efficiency by modifying the data flow of the model rather than by fundamentally redesigning the stereo architecture. Existing efficient stereo networks primarily reduce computational cost through lighter modules, recurrent refinement, or specialized backbones, whereas low-level approaches improve throughput through platform-specific mechanisms such as custom hardware or concurrent execution. The proposed method instead targets a common redundancy in stereo pipelines: the repeated execution of the same feature extractor for the left and right images. By merging this stage into a single pass at the level of the model data flow, the method provides a simple and portable optimization that can be applied to existing and future stereo models while preserving their original architecture and accuracy characteristics.

## 2. Materials and Methods

The downstream matching, cost aggregation, and refinement stages receive tensors with the same structure as in the baseline pipeline. Therefore, the proposed method modifies only the data flow around the FE module and leaves the internal network architecture unchanged.

**Figure 2:**
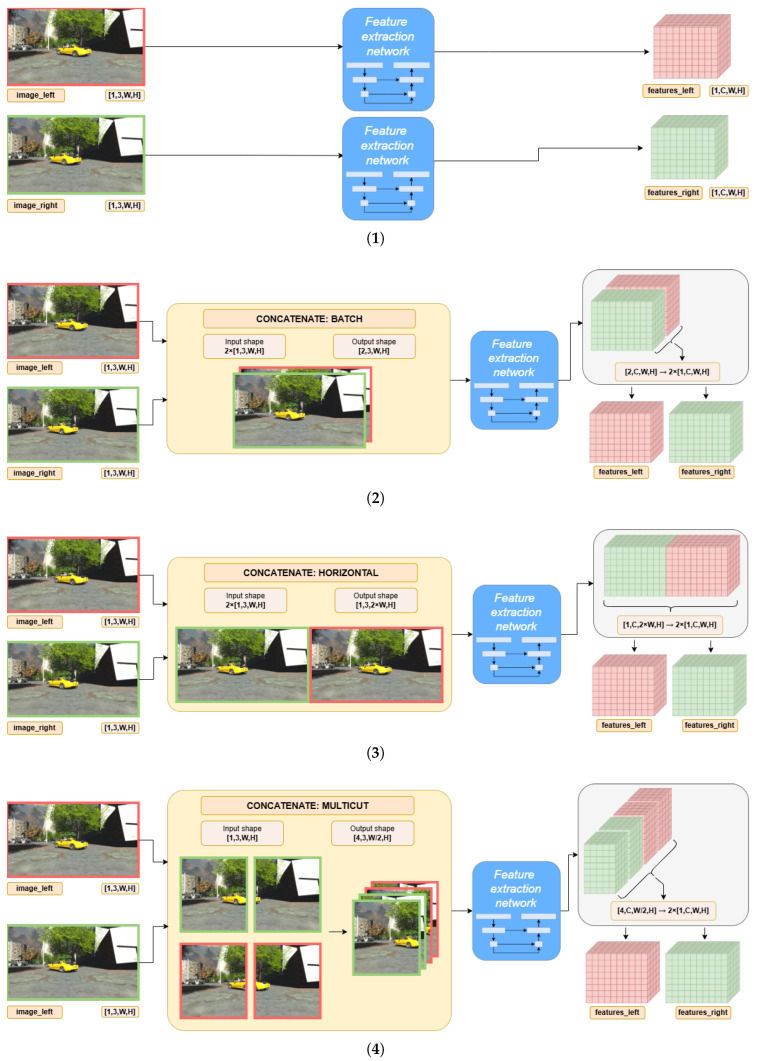
Illustration of different concatenation configurations. Numbers in square brackets present [batch size, number of channels, width, height] of the image or feature map. (**1**) Baseline, labeled as *default*. (**2**) Concatenate along batch, labeled as *a*. (**3**) Concatenated along either width or height dimension (not illustrated), labeled as *b* and *c*. (**4**) Multicut: image is cut horizontally and vertically, then concatenated along batch; *d*: only vertically cut, *e*: both horizontally and vertically, *ea*: no slicing-effectively the same as *a*.

Traditionally, the left and right input images are separately and sequentially processed by the same FE network. In contrast, we concatenate the two images and extract features together from both images in a single forward pass. We test seven concatenation configurations labeled as *default*, *a*, *b*, *c*, *d*, *e*, *ea*, illustrated by the Figure 2 and described in Table 1. The configurations can be divided into four main categories:

1. Baseline: The *default* method represents the original, i.e., unmodified version of each tested model, where FE is implemented as two separate modules that run successively (Figure 2(1)).
2. Batch-based concatenation: In our first proposed method, *a*, we concatenate the image pair along the batch dimension, similarly to how batching is done in batched training (Figure 2(2)). In this method, the image pair stays completely independent of each other. Since batch processing is the universal paradigm for training deep neural networks, the entire ecosystem (from PyTorch down to the GPU hardware) is inherently optimized for it. Thus, we anticipate strong performance improvements with minimal side effects.
3. Spatial concatenation: In the second and third implementations, *b* and *c* (Figure 2(3)), we concatenate the image pair along the same 2D plane in either horizontal or vertical direction, i.e., we double the width or height, respectively.
4. Multi-cut batching: In the next two methods *d* and *e* (Figure 2(4)), we first cut the two images to multiple smaller (sub)images and have them concatenated along the batch dimension. Configuration *d* slices once vertically at the middle of each image, while *e* slices twice–once vertically and once horizontally. Therefore from the two starting images, i.e., left and right, we obtain four or eight subimages, respectively, and concatenate them along the batch dimension. While heavy batching might accelerate inference, the required disassembly and assembly process might, in turn, decelerate it.

We observed that configuration *a* occasionally yields sub-optimal performance for certain FE architectures. To isolate the effect of implementation details, we introduce configuration *ea*, which is functionally equivalent to *a* (batch concatenation) but implemented using the same slicing and reconstruction operations as configurations *d* and *e*. This allows us to evaluate how low-level graph structure affects performance independently of the conceptual concatenation strategy.

Our concatenation methods are implemented as a wrapper around an existing FE module, without modifying its internal architecture. The wrapper provides several forward() implementations, with the appropriate one selected at model build time based on a configuration file. Because these configurations require different input and output tensor shapes, the wrapper also manages the necessary data assembly and disassembly. A formal mathematical description of this single-pass extraction procedure, along with the corresponding tensor dimensions, is provided in Appendix A. Furthermore, for complete implementation details, our source code is publicly available in our GitHub repository.

For our experiments, we use the OpenStereo [23] framework that implements many recently published models for stereo depth inferencing. Furthermore, it provides a convenient environment to modify existing work and implement new architectures. We select three distinct models to demonstrate the general applicability of our proposed modifications: IINet [11] for its recognized efficiency, accuracy, and novel architecture; LightStereo [12] as it is currently one of the fastest models while maintaining good accuracy; and LeanStereo [13], since its FE stage deviates from the classic U-based architectures, thus validating our method on structurally different designs. To evaluate the inference rate, we export PyTorch model to ONNX format and then run it on Nvidia V100 using TensorRT.

### 2.1. Inference Benchmarking

In our first test, we evaluate the modified models in terms of inference speed as this is the main focus of our proposed modifications. We configure the model in PyTorch v2.4.1 and train on a reduced dataset to obtain valid weights. The model is exported in ONNX format (operator set v17), and then, in a new process, the ONNX file is loaded and evaluated using TensorRT framework v8.6.1. and CUDA v12.1. In this experiment, we evaluate relative performance between different configurations. We normalize all results to the baseline to make it clearer how much each configuration affects the execution time. Since our proposed techniques only affect the FE stage, the effective speedup is limited by the proportion of total inference time spent in this stage, as described by Amdahl’s law [24] in Equation (2).

(2) $$S=\frac{1}{\left(1-p\right)+\frac{p}{s}}$$

Configuration *a* is the simplest implementation with a single concatenate operation along batch at the input (left and right image) and a single index slicing operator at the output (feature map). All other implementations require much more slicing and concatenation operations to first construct the concatenated image and later split and re-construct feature maps corresponding to the initial images. We expect that all other implementations will therefore be much slower compared to *a* due to complex disassembling and reassembling processes, yet they should still show a major improvement over the baseline.

Absolute inference speed is influenced not only by model design but also by environment variables, momentary memory consumption, and TensorRT’s internal, non-deterministic kernel selection, all of which cannot be fully controlled by the user. To mitigate these effects and ensure strict isolation, we completely clear the training context and use garbage collection once the model is exported. The model is then loaded in 32-bit floating point and evaluated within a standalone benchmarking process. After an initial warm-up, inference is run on a single input pair for 500 iterations and averaged over six runs, following the approach in [13].

### 2.2. Accuracy Benchmarking

In the second test, we focus on accuracy. When training LightStereo [12], IINet [11] and LeanStereo [13], we follow the authors’ training setup with the exception of LeanStereo where we use OneCycleLR instead of MultiStepLR. To improve training stability, we set gradient norm limiting to 2.0 for IINet and disable AMP for all models. We use batch sizes 24, 16 and 8, and train for 90, 105 and 80 epochs for LightStereo, IINet and LeanStereo respectively. While training on complete datasets with augmentation is standard practice for maximizing absolute accuracy and generalization, our study focuses on a relative comparison to verify that the proposed modifications preserve baseline accuracy. Therefore, we train on the reduced SceneFlow (finalpass) [25] dataset consisting of 50% of all train samples from FlyingThings3D, Monkaa and Driving subsets. Training is done on 320 × 704 images, while evaluation and testing is done on 576 × 960 resolution. To reduce training variability all images are non-augmented, and only normalized using mean and standard deviation values based on ImageNet [26] statistics. The test subset consisted of FlyingThings3D samples only.

Our proposed modifications do not alter the model architecture so we do not expect any significant change in accuracy. However, partitioning the image into sub-regions as in *d* and *e* disrupts the natural receptive fields of kernels along the cut boundaries. Consequently, features near these artificial edges are computed using synthetic padding instead of the true neighboring context, leading to localized accuracy degradation. Therefore, we expect slightly reduced accuracy for these two configurations, although the subsequent refinement stage likely mitigates any localized errors stemming from the FE.

## 3. Results

### 3.1. Inference Rate

**Figure 3:**
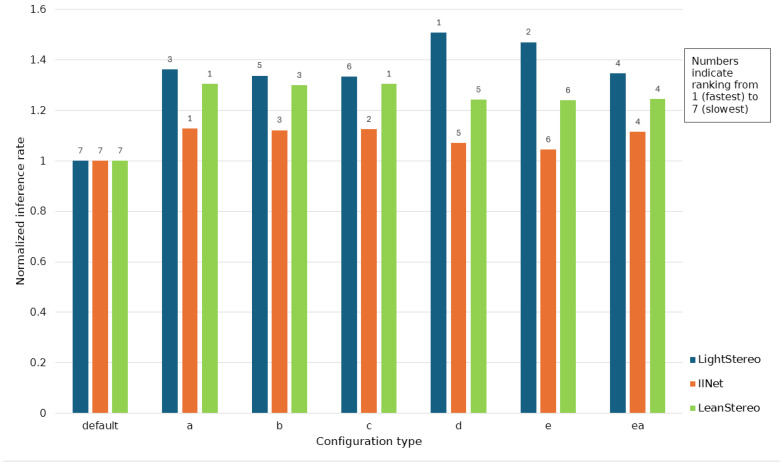
Inference rate for LightStereo, IINet and LeanStereo models normalized to *default* configuration presenting baseline.

Table 2 presents the inference rate in frames per second (FPS), normalized to the baseline (*default*). The same data is illustrated in Figure 3, where the numbers on top of the bars indicate the ranking from fastest (1) to slowest (7). We observe that all of our proposed modifications resulted in a performance gain, although to varying degrees depending on the model. For the LightStereo model, the largest performance gains (51% and 47%) were achieved using the multi-cut configurations *d* and *e*. This is unexpected, as these configurations require complex disassembly and reassembly processes via bitslicing. Profiling results (detailed in the subsequent section) indicate that the benefits of batched inference on smaller input images outweigh the assembly overhead. The remaining configurations *a*, *ea*, *b* and *c* were all quite close. Conversely, configurations *d* and *e* performed the worst for the IINet and LeanStereo models while *a* performed the best, which closely aligns with our initial expectations.

Given that the FE architectures of IINet and LightStereo are highly similar, one would expect their performance profiles to align. Surprisingly, the experimental results for IINet are much more comparable to those of LeanStereo rather than LightStereo, with which it shares many more similarities. This indicates that model design alone cannot explain these conflicting outcomes, suggesting that underlying backend optimization effects play a dominant role. Investigating these interactions remains an important direction for future work.

While the performance improvements in IINet were comparatively modest ranging from 4% to 13% (averaging 10%), the gains for LightStereo and LeanStereo were more substantial, spanning 33% to 51% (averaging 39%) and 24% to 30% (averaging 27%), respectively. Overall, all configurations improve inference speed relative to the baseline across all evaluated models.

While no single configuration consistently outperforms all others, configuration *a* (batch concatenation) provides the most stable and predictable improvements across models. However, configurations *d* and *e* can yield higher gains in certain cases but are more sensitive to model architecture and backend optimization (TensorRT graph generation). Therefore, we encourage researchers to empirically evaluate all configurations to find the optimal one for their specific underlying model.

#### Stage Profiling

We perform layer-level profiling to show how the overall inference time is affected by our modifications. We use TensorRT’s profiling feature and measure across 500 inferences of the LightStereo model. We identified and categorized the reported layers into 7 categories:

- *FE impl.*: The core FE process executed by the network.
- *FE assembly & FE disassembly*: The computational overhead introduced by slicing, concatenating, and reorganizing the input images or resulting feature maps according to the selected configuration.
- *Cost agg.*: The cost aggregation process, which corresponds to the coarse depth estimation stage illustrated in Figure 1.
- *Cost volume*: The construction of the initial cost volume, part of the feature matching stage shown in Figure 1.
- *Refinement*: The post-processing stage where initial, noisy coarse depth estimates are smoothed and enhanced into a coherent depth map.
- *Out stage*: The final processing step that converts the network’s raw numerical outputs into actual pixel-level disparity values.
- *Uncategorized*: Miscellaneous execution nodes within the computation graph that cannot be strictly assigned to any of the defined categories above.

Table 3 shows that FE dominates inference, consuming 54.2% of the total execution time in the *default* implementation. Our modifications reduce this FE duration by 48.4% on average, with only a minor overhead introduced by tensor assembly and disassembly. According to Amdahl’s Law, optimizing a component that accounts for 54.2% of the execution time by reducing its duration by 48.4% yields a theoretical overall execution time reduction of 26.2%, which translates to a total speedup factor of roughly 1.35×. This theoretical expectation aligns with our empirical overall average speedup of 1.39× for LightStereo; the minor discrepancy arises from a slightly different measuring setup between throughput benchmarking and profiling.

FE assembly and disassembly cause additional computational overhead compared to the *baseline*; however, even in the worst case (*e*), they consume less than 3.0% of the total execution time. Out of all configurations, the most computationally demanding are *d* and *e*, which is expected, while the least demanding are *a* and *ea*, which perform direct batching.

Note that TensorRT’s graph optimization occasionally merges the targeted assembly and disassembly operations and reports them under a single category during profiling. Additionally, while the FE output tensor shape remains identical across all configurations, altering the input concatenation type subtly affects the execution times of subsequent network stages due to TensorRT’s non-deterministic graph compilation. Nevertheless, these downstream fluctuations are minimal, confirming that the overall inference speedup is fundamentally driven by the optimized, single-pass FE stage.

### 3.2. Memory Consumption

To provide a comprehensive view of deployment requirements, we report two distinct memory metrics. The model size captures the static, intrinsic footprint of the compiled TensorRT engine primarily consisting of model weights and architectural metadata. We include this metric to show how model size decreases as the exported inference graph now contains a single FE path. Conversely, memory usage measures the aggregate VRAM allocated on the hardware during inference. This metric is crucial because it encompasses the model footprint, data buffers, intermediate execution workspaces, and the baseline CUDA runtime overhead.

Table 4 shows that while the model size decreased, the total required memory increased. This was expected, as the single-pass FE processes twice the data compared to the original, dual-pass approach, making this increase inherently unavoidable. The proposed configurations should be interpreted as a systems optimization: they trade additional transient VRAM for consistent real-time speed gains, and are therefore advantageous whenever memory headroom is available.

### 3.3. Accuracy

To quantify the accuracy of the predicted disparity maps, we utilize the End-Point-Error (EPE) metric which is a standard metric in dense correspondence tasks, such as optical flow and stereo vision, first formalized in [27]. In the case of stereo vision, the equation simplifies to the mean absolute difference:

(3) $$EPE=\frac{1}{N}\sum_{p\in V}\left|d_{est}\left(p\right)-d_{gt}\left(p\right)\right|$$

where *N* is the total number of valid pixels, V is the set of valid pixel locations, $d_{est}\left(p\right)$ is the estimated disparity at pixel *p*, and $d_{gt}\left(p\right)$ is the ground truth disparity at pixel *p*. A lower EPE indicates a more accurate disparity estimation. We evaluate EPE on the test subset of the SceneFlow dataset [25] with samples from only FlyingThings3D.

**Figure 4:**
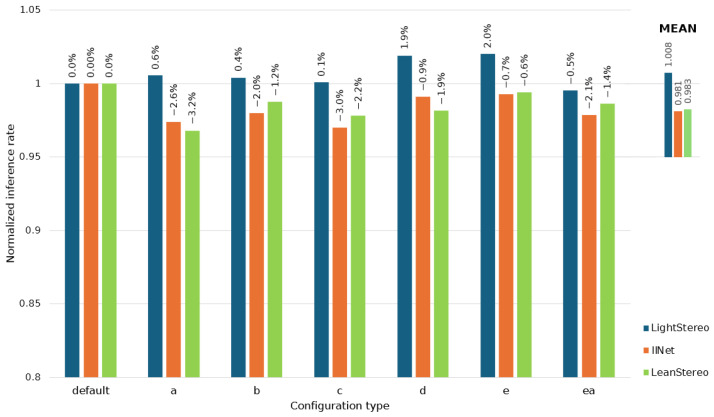
End-Point-Error on test subset for LightStereo, IINet and LeanStereo models normalized to *default* configuration presenting baseline.

Table 5 contains accuracy results normalized to the baseline, and the same data is plotted in Figure 4. We observe that our configurations resulted in a −1.9% and −1.7% lower mean EPE for the IINet and LeanStereo models, respectively, while the EPE for LightStereo slightly increased (+0.8%). We attribute these minor bi-directional fluctuations to two main factors. First, during a combined forward pass, batch normalization parameters are calculated based on joint left and right image statistics. In the original *baseline* implementation with separate FE passes, batch statistics are decoupled. This shift to joint statistics subtly alters the numerical relationships between the stereo pair, which can affect downstream matching accuracy in either direction. Second, the inference framework may dynamically select different convolution algorithms due to the change in input tensor dimensions or batch size. For example, the backend might switch from the Winograd algorithm, which trades numerical precision for speed but restricts tensor shapes, to a more generic General Matrix Multiply (GEMM) algorithm. Ultimately, these systemic factors introduce minor accuracy variations that can be either positive or negative, and they generally fall within the range of expected run-to-run variance.

**Figure A1:**
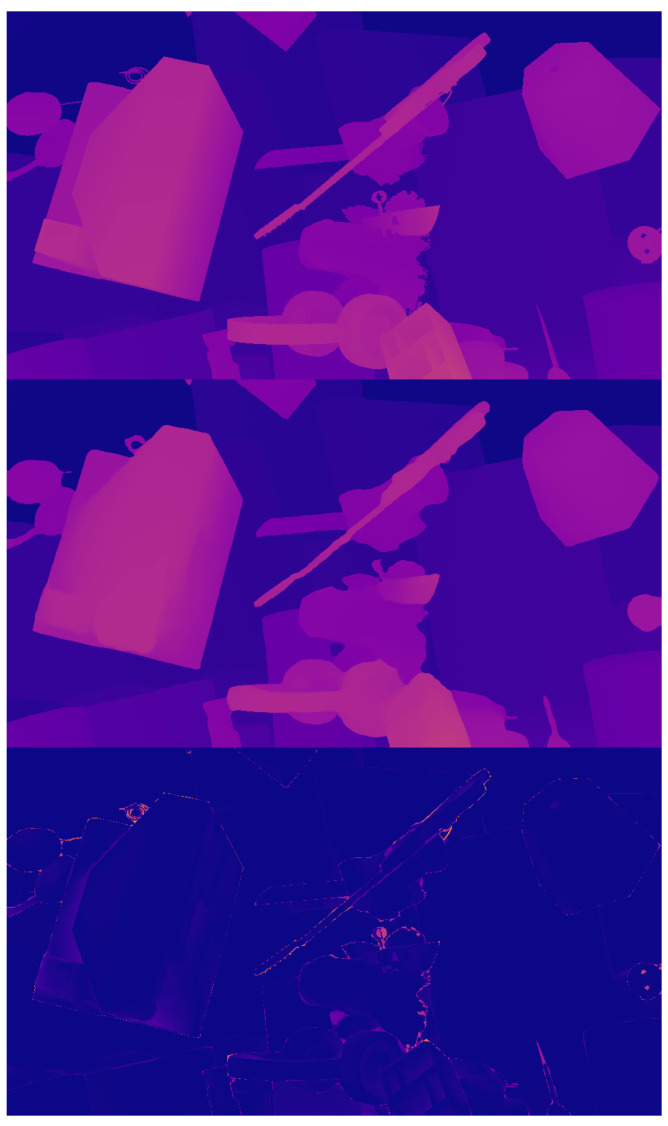
Sample output from LeanStereo model in configuration *e*. From top to bottom: ground truth depth, inferenced depth and error between the two. There are no observable errors along the cutting seams.

Among the proposed configurations, *d* and *e* exhibit slightly lower accuracy. This minor error increase might be related to the artificial cutting seams. However, the resulting localized degradation is not visually apparent, as shown in the error map in Figure A1.

## 4. Edge Device Benchmarking

To demonstrate applicability of our modifications, we perform benchmarking on an edge device. We use OAK 4 D camera from Luxonis, Ljubljana, Slovenia, [28] based on Qualcomm’s QCS8550 SoC (12 TOPS using 16-bit floating point format) with 8GB of RAM for on board processing. It has a monochrome stereo image sensor pair accompanied with high resolution colour image sensor. The manufacturer also provides Robotic Vision Core 4 (RVC4) with development tools. For benchmarking we use modelconverter benchmark command that launches Qualcomm’s Snapdragon Neural Processing Engine (SNPE) benchmarking tools. We used Luxonis OS RVC4 1.31.2 and SNPE v2.32.6. The scripts used for model convertion and benchmarking can be found on our GitHub repository. We ran benchmark with the high_performance SNPE profile setting and utilizing one, two or four threads for inference.

**Figure 5:**
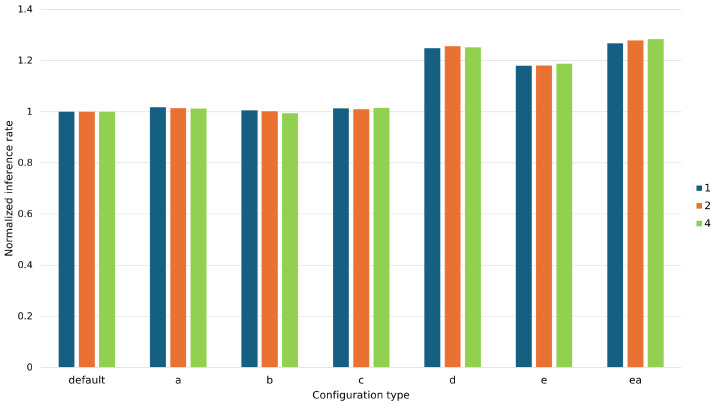
Normalized FPS on edge device (Luxonis OAK 4 D) with 1, 2 and 4 threads active on LightStereo base model.

**Figure 6:**
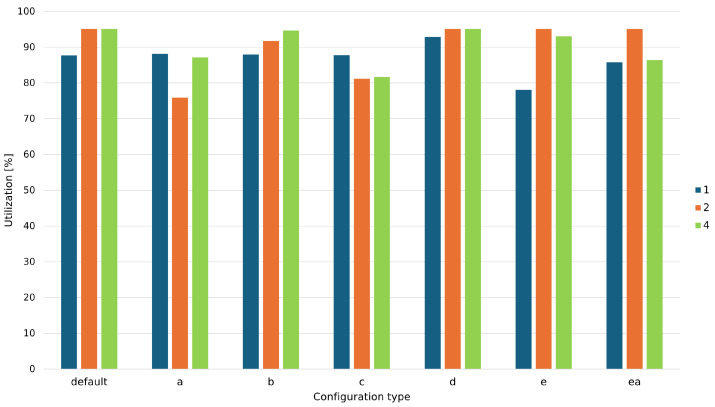
DSP utilization on edge device (Luxonis OAK 4 D) with 1, 2 and 4 threads active on LightStereo base model.

Table 6 shows absolute FPS, FPS normalized to the baseline model, and digital signal processor (DSP) utilization for different numbers of threads. Figure 5 visually presents the normalized FPS data. We observe only a slight advantage for modifications *a*, *b*, and *c*, while modifications *d*, *e*, and *ea* show strong performance gains of 24.8%, 17.0%, and 26.7% in single thread configuration, respectively, while the best gain of 28.4% was achieved in four thread configuration. As explained above, modifications *b* and *c* increase the spatial size of the input by a factor of two, while *d* and *e* decrease the spatial size by factors of two and four, respectively. The results show that, on the edge device, it is more beneficial to decrease the spatial size and stack in the batch dimension than to keep the batch size at 1 and increase the input size. Notably, modification *ea* is conceptually the same as *a*, but a different assembly and disassembly procedure apparently results in better performance. Comparison across number of execution threads shows that performance is largely insensitive to the thread count, indicating that the performance gains do not come from batched or multiprocess execution, but from the fused FE introduced by our work. The Table 6 further shows that mean absolute utilization rates remain approximately constant in the range of 87–90%. This suggests that the proposed modifications more efficiently use available resources, rather than simply increase their utilization. Figure 6 visualises DSP utilization rates.

The tools further enabled us to measure memory consumption. On the OAK 4 D, we measure total system-level usage and per-process usage, which excludes memory consumed by DSP, and report results in Table 7. Across all evaluated configurations, memory usage remains approximately constant, suggesting that the proposed modifications do not introduce additional memory pressure. On the OAK 4 D camera with 8 GB of RAM the observed memory consumption remained well below 20%.

## 5. Discussion

The results demonstrate that eliminating redundant FE passes can significantly improve inference efficiency in stereo depth estimation models. Across all evaluated architectures, the proposed method consistently increases inference speed while maintaining comparable accuracy.

A key advantage of the approach is its simplicity and ease of integration. Since it is implemented as a wrapper around the FE module, it does not require modifications to the internal network architecture, making it applicable to a wide range of existing and new stereo models.

The effectiveness of different concatenation configurations depends on both the model architecture and the inference backend. Batch-based concatenation (configuration *a*) provides the most consistent performance improvements across models. In contrast, multi-cut batching (configurations *d* and *e*) can yield higher speedups in specific cases, but their performance is less predictable and appears to depend on backend-specific optimization behavior.

Furthermore, layer-level profiling explicitly validates the core premise of our approach. The measured speedups are fundamentally driven by a nearly 50% reduction in FE duration, translating to overall inference gains that closely align with theoretical expectations derived from Amdahl’s Law. Notably, the profiling demonstrates that the computational overhead introduced by tensor assembly and disassembly is relatively minor compared to other network stages, consuming less than 3% of the total execution time even in the most complex multi-cut configurations.

The proposed method introduces a trade-off in memory usage. While model size decreases due to reduced duplication in the inference graph, runtime memory consumption increases because both images are processed simultaneously. As a result, the method is most suitable for scenarios where sufficient memory resources are available and inference speed is the primary concern.

These results complement recent efficient stereo networks such as LightStereo and LeanStereo, which improve inference speed mainly through architectural choices such as lightweight backbones or efficient aggregation modules. In contrast, the proposed method preserves the original architecture and merges FE into a single operation at the model data flow level. It is therefore also complementary to runtime-level acceleration strategies presented in [20,22] which improve utilization through deployment-specific scheduling and custom hardware.

Main experiments in this study were conducted on an NVIDIA V100 GPU using TensorRT, while measurements on OAK 4 D edge device with SNPE inference stack confirm practical applicability of our proposed modifications. On the edge device, our multicut modifications resulted in up to 17.9–26.7% higher inference rate compared to the baseline. These results strengthen the central claim that merging separate left/right FE passes is a portable optimization to on edge real time devices. This is because the method is a conceptual data-flow modification around the FE module and does not introduce hardware-specific operators or rely on TensorRT-specific functionality. Therefore, the approach should remain applicable to other deployment targets, including other embedded GPUs and alternative inference runtimes. At the same time, the different behavior of configurations across TensorRT and SNPE shows that backend-specific graph optimization, kernel selection, tensor layout handling, and memory hierarchy still influence the optimal concatenation strategy and its implications. While the proposed method is broadly applicable, it still depends on specific deployment setup. Therefore multicut variants should be considered a promising starting point, but the final decision should be selected based on target platform performance. Further evaluation across additional embedded platforms and inference runtimes accompanied with detailed low-level analysis represents an interesting direction for future work.

## Figures and Tables

**Table 1:** Description of different concatenation configurations used in our experiments.

Configuration Label	Description
default	This is the default model as published by the authors. FE is done sequentially two times. Serves as the baseline in all our experiments.
a	The left and right image are concatenated along batch dimension. FE is done once for both images.
b	The left and right image are concatenated together in vertical direction, i.e., along height dimension. FE is done once.
c	The left and right image are concatenated together in horizontal direction, i.e., along width dimension. FE is done once.
d	The left and right image are cut and halved in width dimension. Then the 4 subimages are concatenated along batch dimension.
e	The left and right image are cut and halved in both height and width dimension. Then the 8 subimages are concatenated along batch dimension.
ea	The left and right image are not cut at all but concatenated along batch dimension. This is effectively the same as configuration *a* but implemented in the same way as *d* and *e*.

**Table 2:** Inference performance comparison. Values represent frames per second (FPS) and relative speedup factor. Average is calculated across all configurations except *default*.

Config	LightStereo		IINet		LeanStereo	
	FPS	Speedup	FPS	Speedup	FPS	Speedup
*default*	137.38	1.00	56.22	1.00	31.83	1.00
*a*	187.13	1.36	63.44	**1.13**	41.53	**1.30**
*b*	183.73	1.34	63.04	1.12	41.39	**1.30**
*c*	183.10	1.33	63.25	**1.13**	41.53	**1.30**
*d*	207.15	**1.51**	60.18	1.07	39.57	1.24
*e*	202.03	1.47	58.73	1.04	39.45	1.24
*ea*	184.98	1.35	62.73	1.12	39.64	1.25
Avg.	191.35	1.39	61.86	1.10	39.28	1.27

**Note:** Bold values indicate the highest speedup achieved for each model.

**Table 3:** Profiling results across different configurations. Values are presented in milliseconds (ms) for 500 inferences, with frames per second (FPS) at the bottom. Profiled on LightStereo model.

Component	*default*	*a*	*b*	*c*	*d*	*e*	*ea*
FE impl.	1822.04	936.36	941.70	962.23	902.97	906.50	982.12
Cost agg.	711.21	738.15	733.61	733.61	675.62	677.16	701.14
Cost volume	468.67	451.77	458.50	453.66	353.20	368.97	575.15
Refinement	281.79	279.70	275.23	288.23	241.46	240.62	245.60
Out stage	45.07	61.45	61.18	61.14	61.35	61.25	61.87
FE assembly	-	-	-	-	36.12	68.33	-
FE disassembly	-	7.59	23.24	25.45	-	-	-
Uncategorized	31.93	47.46	48.17	49.07	28.89	29.19	48.41
Total	3360.70	2522.48	2541.62	2573.38	2299.61	2352.01	2614.29
FPS	148.78	198.22	196.72	194.30	217.43	212.58	191.26

**Table 4:** Model size and runtime memory usage across different configurations.

Config	LightStereo		IINet		LeanStereo	
	Model Size (MB)	Memory Usage (MB)	Model Size (MB)	Memory Usage (MB)	Model Size (MB)	Memory Usage (MB)
*default*	16.34	761.88	48.19	1041.88	28.08	1533.88
*a*	10.25	1197.88	45.57	1375.88	18.90	1909.88
*b*	10.28	1197.88	44.14	1375.88	16.96	1907.88
*c*	10.26	1197.88	45.82	1377.88	18.97	1909.88
*d*	10.28	1141.88	43.30	1389.88	16.91	1641.88
*e*	10.39	1139.88	43.54	1397.88	17.00	1639.88
*ea*	10.31	1135.88	45.44	1409.88	16.91	1643.88
Avg.	10.30	1168.55	44.64	1387.88	17.61	1775.55
	−37.0%	53.4%	−7.4%	33.2%	−37.3%	15.8%

**Table 5:** Accuracy analysis (End-Point Error). Values represent EPE (lower is better) and the relative (<1.0 is better) ratio compared to the default baseline.

Config	LightStereo		IINet		LeanStereo	
	EPE	Ratio	EPE	Ratio	EPE	Ratio
*default*	0.8126	1.000	0.7629	1.000	0.9505	1.000
*a*	0.8171	1.006	0.7429	0.974	0.9202	0.968
*b*	0.8160	1.004	0.7477	0.980	0.9389	0.988
*c*	0.8135	1.001	0.7402	0.970	0.9297	0.978
*d*	0.8279	1.019	0.7560	0.991	0.9329	0.981
*e*	0.8290	1.020	0.7573	0.993	0.9449	0.994
*ea*	0.8089	0.995	0.7466	0.979	0.9376	0.986
Avg.	0.8187	1.008	0.7485	0.981	0.934	0.983
		(+0.8%)		(−1.9%)		(−1.7%)

**Table 6:** Benchmarking on OAK 4 D on LightStereo base model with 1, 2 or 4 execution threads. Values represent FPS, normalized FPS relative to the *default* baseline, and DSP utilization.

Config	FPS			Norm. FPS			DSP Util. [%]		
	1	2	4	1	2	4	1	2	4
*default*	30.17	32.76	32.84	1.000	1.000	1.000	87.75	95.14	95.13
*a*	30.70	33.20	33.24	1.018	1.013	1.012	88.13	75.87	87.17
*b*	30.33	32.81	32.65	1.005	1.002	0.994	87.96	91.76	94.64
*c*	30.56	33.08	33.31	1.013	1.010	1.014	87.80	81.18	81.67
*d*	37.65	41.16	41.12	1.248	1.256	1.252	92.88	95.14	95.12
*e*	35.58	38.70	39.00	1.179	1.181	1.188	78.09	95.11	93.04
*ea*	38.24	41.89	42.16	**1.267**	**1.279**	**1.284**	85.78	95.14	86.38
Avg.	33.32	36.23	36.33	1.104	1.106	1.106	86.91	89.91	90.45

**Note:** Bold values indicate the highest speedup achieved for each number of threads.

**Table 7:** Benchmarking on OAK 4 D on LightStereo base model with 1 thread. Values represent processor memory usage, and DDR memory usage.

Config	Processor Memory [MB]	Total Memory [MB]
*default*	159.27	1350.16
*a*	158.64	1349.04
*b*	158.53	1345.88
*c*	157.54	1346.50
*d*	157.27	1342.24
*e*	157.42	1343.79
*ea*	156.53	1343.89

## Data Availability

The source code used in this study is publicly available on GitHub at https://github.com/joej970/OpenStereo_DoItOnce (accessed on 7 June 2026). The datasets analyzed in this work are publicly available and can be accessed via their respective publishers’ websites.

## Abbreviations

The following abbreviations are used in this manuscript:

AI	Artificial Intelligence
FPGA	Field Programmable Gate Array
FE	Feature Extraction (Network)
SSD	Solid-state Drive
EPE	End-Point-Error
ONNX	Open format to represent neural models [21]
GPU	Graphics Processing Unit
VRAM	Video Random Access Memory on GPU
FPS	Frames Per Second
ARNES	Academic and Research Network of Slovenia
HPC	High-performance Computing
MPS	Nvidia Multi-Process Service
RVC4	Robotic Vision Core 4
OS	Operating System

## Appendix A. Single Pass FE Formulation

Let $I_{L},I_{R}\in R^{B\times 3\times H\times W}$ denote the left and right stereo images. In the baseline stereo pipeline, the feature maps are obtained by two independent, albeit structurally identical, FE networks denoted as $\Phi_{L}\left(\cdot \right)$ and $\Phi_{R}\left(\cdot \right)$:

(A1) $$F_{L}=\Phi_{L}\left(I_{L}\right),\;F_{R}=\Phi_{R}\left(I_{R}\right),\;\Phi_{L}\approx \Phi_{R}.$$

These dual passes are executed sequentially, which duplicates the FE computation for every stereo pair.

The proposed method replaces this operation with an assembly–extraction–disassembly procedure. For a configuration k∈{a,b,c,d,e,ea}, an assembly operator $A_{k}$ first maps the stereo pair to a combined tensor $I_{C}^{\left(k\right)}$:

(A2) $$I_{C}^{\left(k\right)}=A_{k}\left(I_{L},I_{R}\right).$$

A single unified FE network $\Phi_{C}\left(\cdot \right)$ is then evaluated once on the concatenated data:

(A3) $$F_{C}^{\left(k\right)}=\Phi_{C}\left(I_{C}^{\left(k\right)}\right).$$

A disassembly operator $D_{k}$ reconstructs the left and right feature maps:

(A4) $$\left(F_{L}^{\left(k\right)},F_{R}^{\left(k\right)}\right)=D_{k}\left(F_{C}^{\left(k\right)}\right).$$

Finally, let Ψ(·,·) denote the remaining downstream part of the complete neural model (including cost volume construction, aggregation, and disparity refinement). This downstream operator is identical across all modifications, deriving the final predicted disparity map $d_{est}$ from the extracted features:

(A5) $$d_{est}=\Psi \left(F_{L},F_{R}\right)\approx \Psi \left(F_{L}^{\left(k\right)},F_{R}^{\left(k\right)}\right).$$

The dimensions of the assembled input tensor $I_{C}^{\left(k\right)}$ produced by the assembly operator $A_{k}$ for each configuration are summarized in Table A1.

**Table A1:** Dimensions of the assembled input tensor $I_{C}^{\left(k\right)}$ for each configuration.

Configuration (*k*)	Tensor Dimensions (B×C×H×W)
*default*	B×3×H×W (two separate tensors)
*a*, *ea*	2B×3×H×W
*b*	B×3×2H×W
*c*	B×3×H×2W
*d*	4B×3×H×W/2
*e*	8B×3×H/2×W/2
